# A call for multi-agency collaboration in the Pacific Island countries’ mental health – The World Federation for Mental Health viewpoint

**DOI:** 10.1177/10398562241285377

**Published:** 2024-09-25

**Authors:** Neeraj Gill

**Affiliations:** The World Federation for Mental Health, Gold Coast, QLD, Australia; School of Medicine and Dentistry, 97562 Griffith University, Southport, QLD, Australia; Mental Health Policy Unit, Health Research Institute, University of Canberra, Canberra, ACT, Australia

**Keywords:** Pacific Island countries, Western Pacific, mental health, the World Federation for Mental Health

## Abstract

**Objective:**

To introduce the World Federation for Mental Health (WFMH) viewpoint on the Pacific Island countries’ mental health capacity building.

**Conclusion:**

Multi-agency collaboration guided by a nuanced understanding of the local context can enable a public health model of mental health service delivery in the Pacific Island countries.

The Pacific Island countries (PICs) located in the Pacific Ocean include Cook Islands, Fiji, Kiribati, Marshall Islands, Federated States of Micronesia, Nauru, Niue, Palau, Papua New Guinea, Samoa, Solomon Islands, Tonga, Tuvalu, and Vanuatu. While every country is unique in geographical, historical, and cultural contexts, there are shared challenges and priorities in advancing mental health. Across the region, the growing burden of mental health conditions is associated with a rising unmet demand for care. There is an increasing overlap between physical and mental health conditions. Mental health literacy is poor, and stigma, false beliefs, and narratives are prevalent. In addition, small islands in the Pacific Ocean have been deemed to be particularly vulnerable to the direct and indirect impacts of climate change. While natural disasters lead to widespread displacement and trauma, existing and anticipated sea-level rise causes fear and worry on a personal and community level. Mental health is not given priority by most governments in PICs. Resources allocated to mental health are minimal and often centralised in stand-alone mental institutions.^1^ The World Health Organization (WHO) has developed a regional framework for mental health in the Western Pacific. This article highlights the need for collaboration among local, national, and international organisations working to build mental health capacity in the PICs.

## The World Federation for Mental Health

The World Federation for Mental Health (WFMH) is an international membership organisation that was established in 1948 to promote the highest attainable level of mental health for everyone. Its mission is to advance, amongst all people and nations, the promotion of mental health, prevention of mental disorders, and provision of best practice recovery-focused interventions. It is the oldest mental health advocacy organisation and has been working closely with the United Nations (UN) and the World Health Organization (WHO) for decades.^2^ The WFMH has a global membership from all UN and WHO Regions of the world and includes organisations or individuals representing people with lived mental health experience, families, carers, mental health professionals, policy makers, and members of the public with an interest in mental health. The WFMH established the Oceania Mental Health Advisory Committee (OMHAC) in 2022 to strengthen collaboration, advocacy, and strategy on key mental health issues and to promote human rights of populations in the Oceania region, which includes Australia, New Zealand, and the PICs.

## The WHO Regional Framework for mental health in the Western Pacific

The WHO Regional Framework for the future of mental health in the Western Pacific 2023-2030 emphasises the need for promotion of mental health and wellbeing, along with addressing mental health conditions.^3^ This would involve leadership that champions mental health in all policies. In addition, the framework calls for de-centralisation of resources to the community-based ecosystem and primary health care. The WHO Mental Health GAP Action Programme (mhGAP) offers evidence-based guidance to support non-specialist health care providers in low- and middle-income countries in providing care to individuals with mental, neurological, and substance use conditions. In 2019, a regional vision paper, *For the Future: Towards the Healthiest and Safest Region*, was endorsed by Member States during the seventieth session of the WHO Regional Committee for the Western Pacific.^4^ It set four thematic priorities for the Western Pacific to accelerate progress towards the Sustainable Development Goals (SDGs) and universal health coverage: health security; non-communicable diseases (including mental health) and ageing; the health impact of climate change; and reaching the unreached. A WHO progress report of the Western Pacific noted that while significant progress had been made towards the overall SDG3 - ‘good health and well-being’, non-communicable diseases including mental health continued to lag behind.^5^

## Prioritisation of mental health and resource allocation

The public mental health model includes a range of interventions including mental health promotion, prevention of mental disorders, early intervention and treatment, rehabilitation and recovery-oriented support by public mental health services. It is important to ensure that funding for prevention and promotion does not come at the cost of services for severe mental disorders. De-centralisation of resources from stand-alone institutions to community-based mental health services must be done in well-planned and phased manner with appropriate funding and resource allocation. Financial constraints and other competing priorities are often cited as the barriers for mental health reform. Strong advocacy, planning, and expertise are required to persuade and help the political leadership of individual sovereign countries to prioritise mental health. Financial assistance can also be sought from the international funding bodies, for example, the World Bank, the International Monetary Fund, and philanthropic organisations. International and inter-agency collaboration can be the key.

## A call for multi-agency collaboration

The WFMH welcomes the opportunity for local, national, and international organisations to collaborate for mental health capacity building, upskilling, and service delivery in the PICs. The regional priorities must be guided by the grassroot, lived experience of consumers, carers, and mental health professionals in PICs. International organisations like the WFMH, the World Psychiatric Association (WPA), and the World Association of Psychosocial Rehabilitation (WAPR) can collaborate to promote mental health in the Western Pacific. The Royal Australian and New Zealand College of Psychiatrists (RANZCP), Australian College of Mental Health Nurses, New Zealand Nurses Organisation Tōpūtanga Tapuhi Kaitiaki o Aotearoa, Pasifika Medical Association, allied health professional bodies, mental health consumer and carer organisations, and other non-government organisations in Australia, New Zealand, and Pacific Islands for example, TheMHS Learning Network and The Pacific Community should join this committed partnership to work in close collaboration with the WHO regional office for the Western Pacific.

Guided by a nuanced understanding of local contexts, such multi-agency partnerships should support the implementation of the WHO framework through political advocacy and capacity building to enable a public health – whole of community – model of mental health service delivery. This should include increasing mental health literacy; addressing stigma; upskilling primary care workers; and mentorship, education, and support of General Practitioners and other health professionals. Medical, nursing, and allied health university courses must include modules on mental health. These objectives are beyond the remit – and capacity – of any one sector, organisation or agency. The critical element is collective effort – working together. To that end, the WFMH is committed to working collaboratively towards the common goal of supporting wellbeing, and building the capacity of mental health services, across the island nations of the Western Pacific.
